# Fisher’s exact approach for post hoc analysis of a chi-squared test

**DOI:** 10.1371/journal.pone.0188709

**Published:** 2017-12-20

**Authors:** Guogen Shan, Shawn Gerstenberger

**Affiliations:** School of Community Health Sciences, University of Nevada Las Vegas, Las Vegas, NV 89154, United States of America; University of Pittsburgh, UNITED STATES

## Abstract

This research is motivated by one of our survey studies to assess the potential influence of introducing zebra mussels to the Lake Mead National Recreation Area, Nevada. One research question in this study is to investigate the association between the boating activity type and the awareness of zebra mussels. A chi-squared test is often used for testing independence between two factors with nominal levels. When the null hypothesis of independence between two factors is rejected, we are often left wondering where does the significance come from. Cell residuals, including standardized residuals and adjusted residuals, are traditionally used in testing for cell significance, which is often known as a post hoc test after a statistically significant chi-squared test. In practice, the limiting distributions of these residuals are utilized for statistical inference. However, they may lead to different conclusions based on the calculated p-values, and their p-values could be over- o6r under-estimated due to the unsatisfactory performance of asymptotic approaches with regards to type I error control. In this article, we propose new exact p-values by using Fisher’s approach based on three commonly used test statistics to order the sample space. We theoretically prove that the proposed new exact p-values based on these test statistics are the same. Based on our extensive simulation studies, we show that the existing asymptotic approach based on adjusted residual is often more likely to reject the null hypothesis as compared to the exact approach due to the inflated family-wise error rates as observed. We would recommend the proposed exact p-value for use in practice as a valuable post hoc analysis technique for chi-squared analysis.

## 1 Background

This research is motivated by one survey study conducted by Gerstenberger et al. [1] to assess potential influence of introducing zebra mussels to the Lake Mead National Recreation Area (LMNRA), Nevada, USA. Zebra mussels are relative small (finger-nail-sized for adult zebra mussels). Their extremely high reproductive rates raise the concern that they could clog water intakes in the LMNRA as it is the main water resource for the city [2]. They can be easily moved from an affected lake to an unaffected one by attaching to boats, nets, docks, and so on. In this study, surveys approved by United States Fish and Wildlife Service were used to collect data on six different sites in the LMNRA between 2002 and 2003 [1]. All the 274 participants were asked in person about their boating activity types (Pleasure, Angler, Jet Ski, and Other) and their awareness of zebra mussels (Yes/No), see Table 1 for data from this study. The chi-squared test was used to test independence between boater activity type and awareness of zebra mussels, and a very small p-value indicated a strong association between the two factors.

**Table 1:** Awareness of zebra mussels of boaters from Lake Mead National Recreation Area, Nevada, USA.

	Boater activity type				
Awareness	Pleasure	Angler	Jet Ski	Other	Total
Yes	139	15	5	4	163
No	68	15	17	11	111
Total	207	30	22	15	274

Researchers are often interested in identifying significant cells/relationships after a statistically significant chi-squared test [3, 4]. Two test statistics are commonly used to test the significance for each cell. The first test is standardized residual that is calculated as raw residual divided by the squared root of the expected value, where raw residual is defined as the difference between the observed value and the expected value. The second test is adjusted residual: raw residual divided by its standard error. Both tests follow the standard normal distribution asymptotically. These two tests have different conclusions for testing the cells of data in Table 1. In addition to that, statistical inference of these two tests relies on how close the limiting distribution is to the true distribution. For a cell with a relatively small value, asymptotic approaches are often not reliable. Recently, Sharpe [5] reviewed several approaches to conduct a post hoc test after a statistically significant chi-squared test: residual comparison, ransacking, and partitioning. The goal of a post hoc test is to find the source of overall significance.

To overcome the unsatisfactory performance from the existing asymptotic approaches for testing each individual cell in a contingency table after a significant chi-squared test, we propose using Fisher’s approach to compute exact p-value by enumerating all possible tables with the same marginal row and column totals as the observed data. The two aforementioned test statistics can be used to order the sample space, and so does raw residual. It could be very computationally intensive to enumerate all possible tables due to the exponentially increased size of the searching sample space, even with the utilization of efficient numerical search algorithms [6]. For this particular problem, we find that the complete sample space can be reduced to a set of 2 × 2 tables instead of all possible *R* × *C* tables to test the significance of each cell. In addition, we theoretically show that the exact p-values based on the three test statistics are the same, thus they have the same conclusion.

The rest of the article is organized as follows. In Section 2, we review the commonly used approaches to test the significance of each cell after a statistically significant chi-squared test, and propose the exact p-value calculation by using Fisher’s approach. We theoretically prove the relationship between exact p-values based on different test statistics considered in this article. In Section 3, we illustrate the application of the proposed exact p-value by using two real examples including the motivation example from our survey study. We then conduct extensive Monte Carlo simulation studies to compare the performance between the proposed exact approach and the existing asymptotic approaches. Finally, we conclude our research with some remarks in Section 4.

## 2 Methods

In the case that the overall chi-squared test is significant, the next step is to perform a post hoc test to find out which cells from the contingency table are different from their expected values. Without any prior knowledge of each cell, we are interested in testing all cells in a contingency table at once. Three test statistics are often calculated for each cell: Raw Residual (RawR), Standardized Residual (StdR), and Adjusted Residual (AdjR). The larger these residuals are, the greater the contribution of these residuals to the overall chi-squared test.

### 2.1 Residuals

Raw residual is computed as the difference between the observed value and the expected value, which is

$$\begin{array}{c} T_{RawR}=x_{ij}-e_{ij}, \end{array}$$

where *e*_*ij*_ is the expected value of the *ij*-th cell under the independence hypothesis. It has been pointed that the *T*_*RawR*_ is insufficient for hypothesis testing since the *T*_*RawR*_ value tends to be large when the value in that cell is large [5]. For this reason, the following two test statistics were traditionally used for testing independence in the *ij*-th cell. Standardized residual is the component from the chi-squared test, which is

$$\begin{array}{c} T_{StdR}=\frac{x_{ij}-e_{ij}}{\sqrt{e_{ij}}}, \end{array}$$

and adjusted residual uses the standard error of *x*_*ij*_ − *e*_*ij*_ in the test statistic [7, 8]

$$\begin{array}{c} T_{AdjR}=\frac{x_{ij}-e_{ij}}{\sqrt{e_{ij}\left(1-m_{i}/N\right)\left(1-n_{j}/N\right)}}, \end{array}$$

where *m*_*i*_, *n*_*j*_, and *N* are the row marginal total, the column marginal total, and the total sample size, respectively. Both *T*_*StdR*_ and *T*_*AdjR*_ follow the standard normal asymptotically [9].

Both *T*_*StdR*_ and *T*_*AdjR*_ can be used for testing the independence hypothesis for each cell by comparing the calculated test statistics to the critical value from the standard normal distribution. It should be noted that they could reach a different conclusion based on their asymptotic p-values. It is easy to find out that the p-value based on *T*_*AdjR*_ is always less than that based on *T*_*StdR*_, because |*T*_*AdjR*_| is always larger than |*T*_*StdR*_| for an observed data. For this reason, *T*_*AdjR*_ is often recommended for use in practice as compared to *T*_*StdR*_ as the latter test could be too conservative [10].

### 2.2 Exact post-hoc p-value

The accuracy of the limiting distribution for p-value calculation relies on multiple factors: marginal row and column totals, and whether the observed value in that cell is too small. In addition to that, the type I error control by using the limiting distribution is often unsatisfactory [11–17]. To overcome these limitations from using asymptotic approaches for statistical inference, we propose using Fisher’s exact approach in testing the independence. All the possible data with the same marginal row and column totals as the observed data are enumerated and used in the p-value calculation, and the rejection region is determined by using any of the three test statistics: *T*_*RawR*_, *T*_*StdR*_ and *T*_*AdjR*_. Suppose that the marginal row and column totals are *m*_1_, *m*_2_, ⋯, *m*_*R*_, and *n*_1_, *n*_2_, ⋯, *n*_*C*_ in a *R* × *C* contingency table. The probability of observing a data with values **X** = {*x_ij_*, *i* = 1, ⋯, *R*, and *j* = 1, ⋯, *C*} is computed as

$$\begin{array}{c} P\left(X\right)=\frac{\left(m_{1}!m_{2}!\cdots m_{R}!\right)\left(n_{1}!n_{2}!\cdots n_{C}!\right)}{(\prod_{i=1}^{R}\prod_{j=1}^{C}x_{ij}!)N!}, \end{array}$$ (1)

which is often known as the hypergeometric probability. Let *T* be the test statistic to order the sample space, and X* be the observed data. Then, the exact p-value based on Fisher’s approach is calculated as

$$\begin{array}{c} \sum_{X\in \Omega (X^{*})}P\left(X\right), \end{array}$$

where Ω(X*) = {**X** ∶ |*T*(**X**)| ≥ |*T*(X*)|} is the rejection region, and *P*(**X**) is the probability of data **X** as given in Eq (1).

It is very computational to calculate exact p-values without using network search algorithms to find the rejection region effectively. The network algorithm developed by Mehta and Patel [6] has been utilized by many statistical software in computing exact Fisher’s p-value for categorical data that can be organized in a contingency table. Obviously, this algorithm provides a much faster method to find the rejection region than a direct and naive full enumeration which could quickly become impossible as the table size and the total sample size increase. For this particular problem, we can simplify the exact p-value because two data sets having the same *n*_*ij*_ would have the same test statistic. In other words, if a data is in the rejection region, then a set of data that have the same *n*_*ij*_ as that data, should also be in the rejection region. For this reason, the sample space in exact p-value calculation is the collection of data as in Table 2.

**Table 2:** Reorganized data for testing the independence from the *ij*-th cell.

	*C*_*j*_	Other columns combined	Total
*R*_*i*_	*x*_*ij*_	*m*_*i*_ − *x*_*ij*_	*m*_*i*_
Other rows combined	*n*_*j*_ − *x*_*ij*_	*N* − *m*_*i*_ − *n*_*j*_ + *x*_*ij*_	*N* − *m*_*i*_
	*n*_*j*_	*N* − *n*_*j*_	*N*

This new sample size is a collection of data **Y** = (*x_ij_*, *m_i_* − *x_ij_*, *n_j_* − *x_ij_*, *N* − *m_i_* − *n_j_* + *x_ij_*), and the probability of data **Y** is calculated as

$$\begin{array}{c} P\left(Y\right)=\frac{\left[m_{i}!\left(N-m_{i}\right)!][n_{j}!\left(N-n_{j}\right)!\right]}{[x_{ij}!\left(m_{i}-x_{ij}\right)!\left(n_{j}-x_{ij}\right)!\left(N-m_{i}-n_{j}+x_{ij}\right)!]N!}. \end{array}$$

For a 2 by 2 table as in Table 2, it is much easier to enumerate all possible data without the involvement of efficient network search algorithms. Suppose Y* is the observed data. The new exact p-value based on Fisher’s exact approach is computed as

$$\begin{array}{c} \sum_{Y}P\left(Y\right)\times I[|T\left(Y\right)|\geq |T\left(Y^{*}\right)|], \end{array}$$ (2)

where *I*(*a*) is an indicator function with *I*(*a*) = 1 when *a* is true, and zero otherwise.

**Theorem 2.1** *Exact p-value calculations based on the three test statistics are the same*.

*Proof*. The proposed exact p-value by using Fisher’s approach depends on the test statistic *T* to order the sample space. The rejection region is defined as

$$\begin{array}{c} \Psi_{T}\left(Y^{*}\right)=\{Y:|T\left(Y\right)|\geq |T\left(Y^{*}\right)\left|\right\}. \end{array}$$

In the new exact p-value calculation, the row and column marginal totals in Table 2 are considered as fixed. It follows that *e*_*ij*_ and *e*_*ij*_(1 − *m*_*i*_/*N*)(1 − *n*_*j*_/*N*) in the denominate of *T*_*StdR*_ and *T*_*AdjR*_ are constant. Thus, *T*_*StdR*_ and *T*_*AdjR*_ are proportional to *T*_*RawR*_, and it follows that

$$\begin{array}{c} \Psi_{T_{RawR}}\left(Y^{*}\right)=\Psi_{T_{StdR}}\left(Y^{*}\right)=\Psi_{T_{AdjR}}\left(Y^{*}\right). \end{array}$$

By the definition of exact p-value in Eq (2), exact p-values based on these three test statistics are the same for a given data.

We have shown that the three test statistics lead to the same exact p-value from this theorem. They agree with each other for testing individual independence in each cell. For simplicity, we use *T*_*AdjR*_ for sample space ordering to compute exact p-value by using Fisher’s approach.

The classic approach to adjust the significance level for multiple comparisons is the Bonferroni method, which is *α*/*W*, where *W* is the number of comparisons. This correction method is widely used for a problem with independent multiple comparisons. However, in the considered problem for all cells in a contingency table, they are correlated, where the Holm-Bonferroni method can be used. In this method, all *W* p-values are sorted from the smallest to the largest, and the *k*-th smallest p-value is compared with *α*/(*W* + 1 − *k*). This method is uniformly more powerful than the traditionally used Bonferroni method. Later, Simes proposed an improved method for multiple comparisons with the adjusted significance level of *αk*/*W* for the *k*-th smallest p-value [18]. The method by Simes is often more powerful than the two aforementioned methods for multiple comparisons. For this reason, we use the method by Simes for both the asymptotic approach and the proposed approach.

## 3 Results

We first use two real examples to illustrate the application of the proposed exact p-value calculation for a post hoc test after a chi-squared test, then we conduct extensive numerical studies to compare the proposed exact approach with the existing approaches.

### 3.1 Real data application

The first example is a cross-sectional study to study malignant melanoma [19, 20]. In this study, 408 cases were randomly selected from all patients from New South Wales, Australia who was diagnosed with malignant melanoma. Tumor types (4 categories: Hutchinson’s melanotic freckle (H), Indeterminate (I), Nodular (N), and Superficial spreading melanoma (S)) and tumor site (3 categories: Head and neck, Trunk, and Extremities) were recorded for each case. Data of this study is presented in a 4 × 3 contingency table: Table 3. The chi-squared test statistic is calculated as 65.81, with the p-value of 2.9×10^−12^ which is much less than 0.05. Since the overall chi-squared test is significant, we would reject the null hypothesis that tumor type and tumor site are independent.

**Table 3:** Data from the malignant melanoma example for testing independence between tumor type and tumor site.

	Tumor site			
Tumor type	Extremities	Head and neck	Trunk	Total
Hutchinsonś melanotic freckle (H)	10	22	2	34
Indeterminate (I)	28	11	17	56
Nodular (N)	73	19	33	125
Superficial spreading melanoma (S)	115	16	54	185
Total	226	68	106	400

We compute p-values for each cell in this contingency table of this example. First, we use the limiting distributions of test statistics *T*_*StdR*_ and *T*_*AdjR*_ for p-value calculation, see Table 4. This table is sorted by the *T*_*AdjR*_ test statistic from the largest to the smallest. As can be seen from the table, *T*_*StdR*_ is relatively conservative as compared to *T*_*AdjR*_ since *T*_*AdjR*_ has three cells with significant results as compared to one based on *T*_*StdR*_. Suppose *T*_*AdjR*_ is used for statistical inference. We can conclude that the expected count is significantly different from the observed count for tumor types of H at all three tumor sites, and S when head and neck is the tumor site. In addition to these results by using asymptotic approaches, we also provide the proposed exact p-value based on *T*_*AdjR*_ to order the sample space in the last column of Table 4. We have proved in Theorem 2.1 that exact p-values based on the three test statistics are identical. For this particular example, four cells have significant p-values, and the majority of them have tumor type of H at three different tumor sites.

**Table 4:** P-value calculation for each cell of data from the malignant melanoma example. The calculated p-value for each cell is compared to the multiple comparison correction method by Simes [18]. The cells with significant p-values are bold.

								Exact P-value
Site	Type	Freq	*T*_*RawR*_	*T*_*StdR*_	P-value	*T*_*AdjR*_	P-value	*T*_*AdjR*_
Head neck	H	22	263.09	45.52	**1.51**×10^−11^	59.93	**9.77**×10^−15^	**5.62**×10^−11^
Head neck	S	16	238.70	7.59	**5.87**×10^−3^	17.01	**3.71**×10^−5^	**4.91**×10^−5^
Extremities	H	10	84.82	4.42	3.56×10^−2^	11.09	**8.66**×10^−4^	**1.03**×10^−3^
Trunk	H	2	49.14	5.45	1.95×10^−2^	8.11	**4.40**×10^−3^	**3.62**×10^−3^
Extremities	S	115	109.73	1.05	3.06×10^−1^	4.49	3.41×10^−2^	4.29×10^−2^
Trunk	S	54	24.75	0.50	4.77×10^−1^	1.28	2.58×10^−1^	3.07×10^−1^
Extremities	I	28	13.25	0.42	5.18×10^−1^	1.12	2.90×10^−1^	3.11×10^−1^
Trunk	I	17	4.67	0.31	5.75×10^−1^	0.50	4.81×10^−1^	5.14×10^−1^
Head neck	N	19	5.06	0.24	6.25×10^−1^	0.42	5.18×10^−1^	5.68×10^−1^
Extremities	N	73	5.64	0.08	7.77×10^−1^	0.27	6.05×10^−1^	6.64×10^−1^
Head neck	I	11	2.19	0.23	6.31×10^−1^	0.32	5.70×10^−1^	7.02×10^−1^
Trunk	N	33	0.02	0.00	9.83×10^−1^	0.00	9.76×10^−1^	1.00

We revisit the awareness survey in Introduction section as the second example. This personal interview survey data is presented in Table 1, and the overall p-value to test the independence between boater activity type and awareness of zebra mussels in the Lake Mead is calculated as 1.4 × 10^−5^, which indicates a significant association between boater activity type and awareness of zebra mussels. Following a significant chi-squared test, we compute the three test statistics, asymptotic p-values based on *T*_*StdR*_ and *T*_*AdjR*_, and the proposed exact p-value, see Table 5. No significant cell is found by using *T*_*StdR*_, while boaters for pleasure, Jet ski, or other are shown to be significant by using either *T*_*AdjR*_ or the exact approach. In this example, a few observations have the same cell p-values. For such cases, we use the largest adjusted p-value for those having the same p-value. In this example, *T*_*AdjR*_ and the proposed exact approach for p-value calculation have the same conclusion. It should be noted that when a factor only has 2 levels (the awareness in this example, *j* = 1, 2), *T*_*AdjR*_ is the same within each level of the other factor (*T*_*AdjR*_(*x*_*i*1_) = *T*_*AdjR*_(*x*_*i*2_)) [9]. This leads to the same exact p-values for the these two cells as observed in the table.

**Table 5:** P-value calculation for each cell of data from the survey for the awareness of zebra mussels. The calculated p-value for each cell is compared to the multiple comparison correction method by Simes [18]. The cells with significant p-values are bold.

								Exact P-value
Site	Type	Freq	*T*_*RawR*_	*T*_*StdR*_	P-value	*T*_*AdjR*_	P-value	*T*_*AdjR*_
Pleasure	Yes	68	251.47	3.00	0.08	20.61	**5.62**×10^−06^	**7.69**×10^−06^
Pleasure	No	139	251.47	2.04	0.15	20.61	**5.62**×10^−06^	**7.69**×10^−06^
Jet Ski	Yes	5	65.41	5.00	0.03	13.41	**2.50**×10^−04^	**3.11**×10^−04^
Jet Ski	No	17	65.41	7.34	0.01	13.41	**2.50**×10^−04^	**3.11**×10^−04^
Other	Yes	4	24.24	2.72	0.10	7.09	**7.74**×10^−03^	**1.26**×10^−02^
Other	No	11	24.24	3.99	0.05	7.09	**7.74**×10^−03^	**1.26**×10^−02^
Angler	Yes	15	8.10	0.45	0.50	1.26	2.62×10^−01^	3.25×10^−01^
Angler	No	15	8.10	0.67	0.41	1.26	2.62×10^−01^	3.25×10^−01^

### 3.2 Simulation study

We conduct an extensive simulation study to further compare the existing asymptotic approach based on *T*_*AdjR*_ and the proposed exact approach. It has been observed that the asymptotic approach based on *T*_*StdR*_ is relatively conservative as compared to that based on *T*_*AdjR*_. For this reason, we exclude *T*_*StdR*_ in the comparison.

For a given total sample size (*N*) and the size of table (*R* × *C*), we first simulate the row and column marginal totals, (*m*_1_, ⋯, *m*_*R*_) and (*n*_1_, ⋯, *n*_*C*_). We simulate 1,000 sets of the marginal totals. For each simulated marginal totals, we then use an R function, *r*2*dtable*, to randomly generate 2,000 *R* × *C* contingency tables by using Patefield’s algorithm [21, 22]. For each simulated data from these 2,000 tables, we compute the asymptotic p-value based on the limiting distribution of *T*_*AdjR*_ and the exact p-value. We compute the family-wise error rate (FWER) for each approach when performing *R* × *C* hypotheses at the same time for each simulated data. The FWER is calculated as the average of the number of tables whose hypotheses are rejected from at least one cell. The significance level is set as 0.05*k*/(*R* × *C*), *k* = 1, 2, ⋯, *R* × *C* by using the Simes correction method for multiple comparisons.

**Fig 1:**
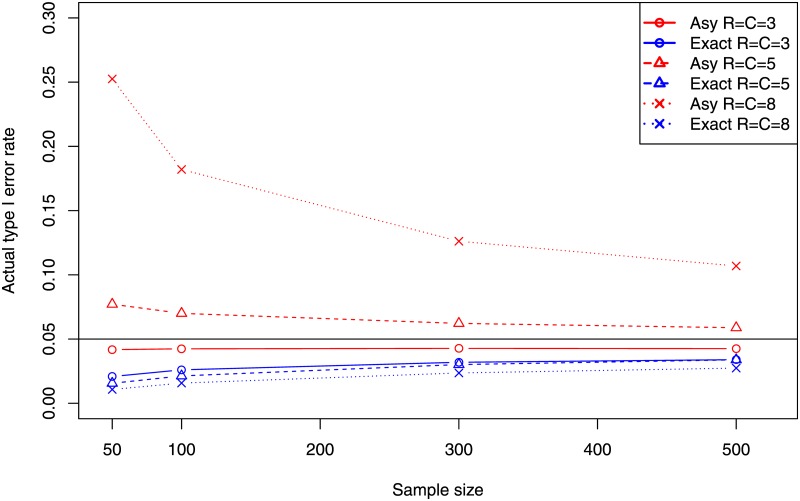
Actual family-wise error rates of the proposed exact approach and the existing asymptotic approach based on the adjusted residual at the nominal level of 0.05.

Fig 1 shows the FWERs for both asymptotic and exact approaches for a contingency table size with sizes of 3 × 3, 5 × 5, and 8 × 8, and sample sizes from 50 to 500. It can be seen that the asymptotic approach does not guarantee the type I error in the majority of cases, and it is almost 5 times the nominal level in one case. The performance of the asymptotic approach gets worse as the size of table increases. It could be caused by the reason that the chance of rejecting at least one of the null hypotheses is increased when more hypotheses are tested simultaneously. The proposed exact approach guarantees the type I error rate.

Suppose Γ_*Asy*_ and Γ_*Exact*_ are the numbers of cells with significant p-values by using the asymptotic approach and the exact approach, respectively. We include the cases that have at least one cell being significant based on one of the two approaches, max(Γ_*Asy*_, Γ_*Exact*_) > 0. In other words, the cases with Γ_*Asy*_ = 0 and Γ_*Exact*_ = 0 are excluded in the performance comparison.

In Table 6, we compare the existing asymptotic approach based on *T*_*AdjR*_ and the proposed exact approach by using all cases with max(Γ_*Asy*_, Γ_*Exact*_) > 0 for given *N* and the table size (*R* = 3 and *C* = 3). The last row of this table shows the total number of such cases from the total 1,000× 2,000 = 2,000,000 simulated data. We find that the proportion of the two approaches having the same conclusion Γ_*Asy*_ = Γ_*Exact*_, increases as the total sample size goes up, and the proportion of Γ_*Asy*_ > Γ_*Exact*_ (the number of cell rejected by the asymptotic approach is more than that by using the exact approach), is a decreasing function of *N*. Among the cases with Γ_*Asy*_ > Γ_*Exact*_, the majority of them are the ones that the exact approach has no significant p-value from any cell. The number of cases such that the exact approach has more rejected cells than the asymptotic approach, is relatively low, which is less 0.15% for the cases studied. When *N* is small, such as *N* = 50, the asymptotic approach always rejects at least the same number of cells as the exact approach, Γ_*Asy*_ ≥ Γ_*Exact*_.

**Table 6:** For a 3 × 3 contingency table, frequency (Freq) and proportion (Prop) of simulated data having at least one cell is significant based on either *T*_*AdjR*_ or exact p-value, from a total of 2 million simulations. Γ_*Asy*_ and Γ_*Exact*_ are the number of cells with significant p-values by using the asymptotic approach and the exact approach, respectively.

	N = 50		N = 100		N = 300		N = 500	
	Freq	Prop	Freq	Prop	Freq	Prop	Freq	Prop
Γ_*Asy*_ = Γ_*Exact*_ > 0	28975	34.69	38868	45.79	52616	61.50	58046	68.15
Γ_*Asy*_ > Γ_*Exact*_ = 0	41845	50.10	32801	38.64	21705	25.37	17256	20.26
Γ_*Asy*_ > Γ_*Exact*_ > 0	12700	15.21	13133	15.47	11136	13.02	9761	11.46
Γ_*Exact*_ > Γ_*Asy*_ = 0	0	0.00	74	0.09	82	0.10	77	0.09
Γ_*Exact*_ > Γ_*Asy*_ > 0	2	0.00	12	0.01	13	0.02	30	0.04
Total	83522	100	84888	100	85552	100	85170	100

We present the frequency and proportion of simulated data from a 3 × 5 contingency table in Table 7 and a 5 × 5 contingency table in Table 8. When the total sample size is small, the proportion of Γ_*Asy*_ = Γ_*Exact*_ is less than that of Γ_*Asy*_ > Γ_*Exact*_, and this trend is reversed as the sample size increases. As the table size increases, the proportion of two approaches having different numbers of rejected cells (Γ_*Asy*_ ≠ Γ_*Exact*_), goes up. Similar to Table 6, these two tables show that the proportion of Γ_*Asy*_ > Γ_*Exact*_ is relatively large as compared to that of Γ_*Asy*_ < Γ_*Exact*_.

**Table 7:** For a 3 × 5 contingency table, frequency (Freq) and proportion (Prop) of simulated data having at least one cell is significant based on either *T*_*AdjR*_ or exact p-value, from a total of 2 million simulations. Γ_*Asy*_ and Γ_*Exact*_ are the number of cells with significant p-values by using the asymptotic approach and the exact approach, respectively.

	N = 50		N = 100		N = 300		N = 500	
	Freq	Prop	Freq	Prop	Freq	Prop	Freq	Prop
Γ_*Asy*_ = Γ_*Exact*_ > 0	27157	27.72	39202	39.40	54207	55.71	60262	62.71
Γ_*Asy*_ > Γ_*Exact*_ = 0	64211	65.53	51568	51.83	34503	35.46	28220	29.37
Γ_*Asy*_ > Γ_*Exact*_ > 0	6614	6.75	8427	8.47	8269	8.50	7373	7.67
Γ_*Exact*_ > Γ_*Asy*_ = 0	0	0.00	268	0.27	267	0.27	196	0.20
Γ_*Exact*_ > Γ_*Asy*_ > 0	0	0.00	23	0.02	58	0.06	42	0.04
Total	97982	100	99488	100	97304	100	96093	100

**Table 8:** For a 5 × 5 contingency table, frequency (Freq) and proportion (Prop) of simulated data having at least one cell is significant based on either *T*_*AdjR*_ or exact p-value, from a total of 2 million simulations. Γ_*Asy*_ and Γ_*Exact*_ are the number of cells with significant p-values by using the asymptotic approach and the exact approach, respectively.

	N = 50		N = 100		N = 300		N = 500	
	Freq	Prop	Freq	Prop	Freq	Prop	Freq	Prop
Γ_*Asy*_ = Γ_*Exact*_ > 0	27316	17.71	37304	26.62	54315	43.55	61890	52.49
Γ_*Asy*_ > Γ_*Exact*_ = 0	123066	79.79	97514	69.58	64434	51.66	50217	42.59
Γ_*Asy*_ > Γ_*Exact*_ > 0	3853	2.50	5167	3.69	5565	4.46	5445	4.62
Γ_*Exact*_ > Γ_*Asy*_ = 0	0	0.00	148	0.11	329	0.26	278	0.24
Γ_*Exact*_ > Γ_*Asy*_ > 0	0	0.00	10	0.01	81	0.06	73	0.06
Total	154235	100	140143	100	124724	100	117903	100

When we compare the three tables in Eqs (6), (7), and (8) with different table sizes, we find that the proportion of max(Γ_*Asy*_, Γ_*Exact*_) > 0 among the total 2 million simulations, is increased as the table size increases. Within the 3 × 5 or 5 × 5 contingency table, the proportion of max(Γ_*Asy*_, Γ_*Exact*_) > 0 is a decreasing function of *N*, while in Table 6 for a 3 × 3 contingency table, this proportion is almost constant across different total sample sizes.

## 4 Discussion

It is well known that asymptotic approaches could lead to different conclusions based on their limiting distributions for p-value calculation. In this article, we theoretically prove that exact p-values produce the same result by using any of the three commonly used test statistics. For this reason, we would like to recommend the proposed exact p-value for use in practice. We develop the software program to compute exact p-value by using the statistical software R [23], and it is available from the first author’s website at: https://faculty.unlv.edu/gshan/ under the Software development section. In addition to that, we also provide a website for researchers who do not use R, which is: http://gshan.i2.unlv.edu/ZPostHoc. We would appreciate any comments from users to further improve the R function and the website.

We do not find an alternative approach based on the exact framework. The existing approaches are generally based on asymptotic limiting distributions or simulation. For the approach based on simulation, it can only simulate a certain number of cases, and it may delete some cases (e.g., the ones with one or more zeros in the table). Although simulation is an approach to utilize when it is difficult to enumerate all possible samples, especially for a study with the total sum fixed [24–27].

In addition to the considered three test statistics for testing cells, several other approaches were developed after a significant chi-squared test. Partitioning is one of them, and this approach basically divide a contingency table into a set of 2 × 2 tables. Obviously, the total possible number of set is $\left(\begin{array}{c} R\\ 2 \end{array}\right)$ × $\left(\begin{array}{c} C\\ 2 \end{array}\right)$. Due to the large number of partitioning, a set of orthogonal partitions was proposed [28] to avoid having too many unnecessary comparisons [5, 29]. Alternatively, Jin and Wang [30] suggested to implement multiple comparisons on one factor. When that factor has *R* levels, each data for a post hoc test is a 2 × *C* contingency table. Then, the total number of comparisons is $\left(\begin{array}{c} R\\ 2 \end{array}\right)$. They compute p-value for each 2 × *C* contingency table by using the chi-squared test. One can always consider using exact approaches for p-value calculation for such data [13]. We consider this as future work.

## 5 Conclusions

In this article, we propose using Fisher’s approach to compute exact p-value for each cell in a contingency table after a significant overall chi-squared test [31–35]. The existing approaches are often based on asymptotic limiting distributions of their associated test statistics. From our extensive simulation studies conducted in this article, we find that the FWERs of the asymptotic approach based on *T*_*AdjR*_ could be much larger than the nominal level, while the proposed exact approach guarantee the FWER. Due a lack of an existing approach with the FWER guaranteed, we do not have another approach to be included to compare with the proposed exact approach with regards to power.
